# Association Between Maternal Angiogenic Markers in Hypertensive Disorders of Pregnancy and Neonatal Growth and Body Composition

**DOI:** 10.3390/nu18162653

**Published:** 2026-08-14

**Authors:** Nawa Schirwani-Hartl, Julia Binder, Pilar Palmrich, Nicholas Longford, Elisabeth Calek, Karin Harreiter, Alexandra Thajer, Angelika Berger, Herbert Kiss, Christoph Binder

**Affiliations:** 1 Department of Obstetrics and Gynecology, Division of Obstetrics and Fetomaternal Medicine, Medical University of Vienna, 1090 Vienna, Austria; 2 Mohn Centre for Children’s Health and Wellbeing, School of Public Health, Imperial College London, London SW10 9NH, UK; 3 Comprehensive Center for Pediatrics, Department of Pediatrics and Adolescent Medicine, Division of Neonatology, Intensive Care and Neuropediatrics, Medical University of Vienna, 1090 Vienna, Austriaalexandra.thajer@meduniwien.ac.at (A.T.);

**Keywords:** hypertensive disorders of pregnancy, sFlt-1/PlGF, body composition, preterm birth, angiogenic markers, fat-free mass

## Abstract

**Background/Objectives:** Hypertensive disorders of pregnancy (HDPs) such as preeclampsia are associated with adverse maternal and perinatal outcomes and related to high soluble fms-like tyrosine kinase-1 (sFlt-1) levels and low placental growth factor (PlGF) levels. While these biomarkers are used for predicting and diagnosing preeclampsia, their impact on postnatal neonatal nutritional management, growth, and body composition is not well-established. Therefore, we aimed to evaluate the association between maternal angiogenic markers, neonatal growth, and body composition. **Methods**: This study represents a secondary analysis of a prospective cohort study conducted at the Medical University of Vienna. Women with HDP were included in the analysis, and infants were stratified by gestational age at birth into preterm (<37 weeks of gestation) and term (≥37 weeks of gestation) groups. Maternal angiogenic markers were routinely measured at the time when HDP was first suspected, and neonatal body composition was assessed at term-equivalent age. **Results**: A total of 335 infants were screened, 94 of which (preterm: *n* = 72; term: *n* = 22) were included. Higher sFlt-1/PlGF ratios were significantly correlated with lower fat-free mass (FFM) z-scores (rs = −0.408; *p* = 0.004), but not with fat mass (FM) z-scores (*p* = 0.21), weight (*p* = 0.19), length (*p* = 0.31), or head circumference z-scores (*p* = 0.32) in preterm infants. In a linear regression model adjusted for potential neonatal confounders, higher sFlt-1/PlGF ratios were independently and significantly associated with lower FFM in preterm infants (median: −0.10, 95% CI: −0.2, 0.00; *p* = 0.036). In term infants, sFlt-1/PLGF ratios were not significantly correlated with FFM (*p* = 0.86), FM z-scores (*p* = 0.41), weight (*p* = 0.10), length (*p* = 0.12), or head circumference z-scores (*p* = 0.2). **Conclusions**: These findings suggest that the sFlt-1/PLGF ratio is not only a well-established marker of HDP severity during pregnancy but may also be associated with adverse effects on body composition in preterm infants, particularly in fat-free mass (FFM).

## 1. Introduction

Women who develop hypertensive disorders of pregnancy (HDPs) are at an increased risk of future cardiovascular diseases, including metabolic syndrome, hypertension, stroke, and ischemic heart disease [1,2]. Among the proposed hypotheses for HDP, including gestational hypertension and preeclampsia, the ischemic model is the most widely recognized, suggesting that reduced uteroplacental perfusion leads to placental hypoxia and subsequent symptoms of placental dysfunction including intrauterine growth restriction and low birth weight [3].

Women with HDP exhibit elevated levels of antiangiogenic factors, such as soluble fms-like tyrosine kinase 1 (sFlt-1), which binds placental growth factor (PlGF) and contributes to systemic endothelial dysfunction, leading to the clinical symptoms of the disease [4,5,6,7,8,9]. Specifically, maternal circulating sFlt-1 levels are typically elevated, whereas PlGF levels are reduced. These angiogenic imbalances are closely associated with disease severity, particularly in early-onset preeclampsia [10,11,12]. This dysregulation of angiogenic factors not only predicts adverse perinatal outcomes but is also strongly linked to an increased long-term cardiovascular risk in both mothers [13,14,15] and their offspring [16,17]. Furthermore, HDP knowingly affects fetal and neonatal growth and body composition [18,19,20]. In a previous study, we found that body composition, particularly fat-free mass (FFM) at term-equivalent age, which is associated with impaired brain growth and neurodevelopment, was lower in preterm infants born to mothers with HDP compared to those without HDP [18]. Although the importance of maternal angiogenic markers during pregnancy is well-established and these markers are widely used for disease diagnosis, their role in determining fetal and neonatal growth and body composition remains unclear and has not been thoroughly investigated.

While assessing growth alone, particularly anthropometric parameters, is insufficient to evaluate nutritional status, evaluating body composition offers a more comprehensive understanding of postnatal nutritional status and allows for assessing nutritional management in infants [21,22]. Neonatal body composition and fetal growth are affected by a complex interplay of fetal, maternal, and placental factors. Established determinants include gestational age at birth and infant sex, as well as fetal growth status, especially small for gestational age (SGA), and modifiable maternal factors, such as maternal diet, metabolic health during pregnancy, and pregnancy complications [23]. Prenatal factors, including maternal HDP, may influence the health of the newborn throughout the life course [24,25]. While fetal growth restriction (FGR) is a well-established consequence of placental dysfunction, new evidence suggests that placental angiogenic imbalance may also impact fetal tissue accretion through impaired placental vascular function, potentially modifying neonatal fat and lean mass independently of birth weight or growth restriction [26,27,28]. However, evidence regarding the relationship between maternal angiogenic markers and neonatal body composition remains limited. Therefore, the aim of this study was to examine the association between maternal angiogenic markers, neonatal growth, and body composition in preterm and term infants at term-equivalent age.

## 2. Materials and Methods

### 2.1. Study Design

This is a secondary analysis of a prospective cohort study [29] conducted at the Department of Obstetrics and Gynecology, Division of Obstetrics and fetomaternal Medicine, and the Department of Pediatrics and Adolescent Medicine, Division for Neonatology Paediatric Intensive Care and Neuropediatrics, at the Medical University of Vienna between July 2019 and September 2023 (HyperDiP study: Maternal hemodynamics in Hypertensive Disorders of Pregnancy under antihypertensive therapy). The HyperDiP study was a prospective observational case–control study evaluating maternal hemodynamic function in women affected by HDP under antihypertensive treatment compared to a healthy control group. The study protocol and study results have been previously published in detail [29,30], where singleton pregnancies affected by HDP and normotensive women (control group) were included. Maternal angiogenic markers were routinely measured at the time when HDP was first diagnosed. Serum concentrations of angiogenic biomarkers were quantified using electrochemiluminescence immunoassays on the Elecsys platform (Roche Diagnostics, Penzberg, Germany). All laboratory procedures were carried out by qualified laboratory personnel.

Neonatal short-term outcomes, anthropometric growth parameters during the hospital stay, nutritional management, and body composition measurements at term age were analyzed in infants born to mothers with HDP. Preterm infants (<37 weeks of gestation) and infants born at term (≥37 weeks of gestation) were analyzed separately. The primary objective of this study was to assess the correlation between maternal angiogenic markers (sFlt-1, PlGF, and their ratio) and the growth and body composition of offspring born to women with HDP. The sample size was enhanced by including preterm infants born between November 2017 and June 2019, who had their body composition routinely assessed according to the local standard protocol, which was introduced in November 2017 in all preterm infants at term-equivalent age. The study was approved by the local ethics committee of the Medical University of Vienna (EK 1882/2018 and 1143/2017) and was conducted according to the Helsinki Declaration of 2013.

### 2.2. Population

The study population comprised women with HDP, including gestational hypertension and preeclampsia, and were recruited from the outpatient clinic of the Department of Obstetrics and Gynecology, Division of Obstetrics and fetomaternal Medicine at the Medical University of Vienna. Inclusion was restricted to women whose infants underwent body composition measurement at term-equivalent age (detailed description provided in the following section). HDP was diagnosed in accordance with the International Society of Hypertensive Disorders in Pregnancy 2021 revised criteria: (1) Gestational hypertension was defined as new-onset systolic blood pressure >140 mmHg and/or diastolic blood pressure >90 mmHg after 20 weeks of gestation in the absence of maternal organ and uteroplacental dysfunction. (2) Preeclampsia was defined as de novo hypertension after 20 weeks of gestation accompanied by one or more of the following: proteinuria (>300 mg/24 h or dipstick reading of >2+), maternal organ failure (liver involvement, renal impairment, hematological or neurological complications), or uteroplacental dysfunction (such as fetal growth restriction) [31]. Angiogenic markers, including sFlt-1 and PlGF, were measured and their ratio calculated as part of the standardized routine clinical assessment of these women. The correlation between angiogenic markers and neonatal outcomes, particularly growth and body composition, was evaluated. Offspring were stratified into two groups: (1) infants born preterm with a gestational age of <37 weeks of at birth and (2) those born at term defined as ≥37 weeks of gestation at birth. Infants with genetic and metabolic disorders and those born to women without HDP were excluded from the study. To evaluate the impact of HDP severity on infant FFM and FM, maternal sFlt-1/PlGF ratios were dichotomized using a clinically validated threshold of 38 [32]. Preterm infants were stratified into two distinct cohorts based on maternal biomarker status: a low-severity group (sFlt-1/PlGF ratio <38) and an elevated-severity group (sFlt-1/PlGF ratio ≥38). This classification of disease severity was included as a covariate in the adjusted multivariable linear regression models to evaluate their independent association with FFM and FM.

### 2.3. Growth and Body Composition Measurements

Anthropometric parameters including weight, length, and head circumference were assessed at birth, during hospital stay, at discharge, and when body composition was measured. Body weight was measured daily using an electronic scale, with values documented at 48 h intervals after the infants reached 1000 g. Body length was assessed with an infant’s length board and head circumference with flexible measuring tape.

Body composition was assessed in term infants and infants born preterm by air-displacement plethysmography (PEA POD^®^ device; COSMED, Concord, CA, USA) between 37 and 44 weeks of postmenstrual age. The method, a non-invasive technique measuring FFM and fat mass (FM), takes about 5 min. The validity and reproductivity have been previously reported [33]. FFM z-scores and FM z-scores were derived using published reference charts stratified by sex and gestational age for preterm infants [34]. Newborns with congenital abnormalities or genetic and metabolic disorders, as well as twins and triplets, were excluded.

The primary outcome of this study was to assess the correlation between maternal angiogenic markers (sFlt-1, PlGF, and their ratio) and FFM in offspring born to women with HDP.

### 2.4. Short-Term Neonatal Outcome Parameters

Retinopathy of prematurity (ROP) was defined according to the International Committee for the Classification of ROP [35], and intraventricular hemorrhage (IVH) was defined according to the criteria published by Papile et al. [36]. The diagnosis of bronchopulmonary dysplasia (BPD) was based on the requirement for supplementation oxygen >21% at 36 + 0 weeks of gestation [37]. Necrotizing enterocolitis (NEC) was classified according to modified criteria established by Bell et al. [38,39]. Illness severity was assessed by SNAPPE-II (Score for Neonatal Acute Physiology with Perinatal extension-II) [40]. Collected demographic data comprised gestational age at delivery, exposure of antenatal corticosteroids, occurrence of preterm premature rupture of the membranes (pPROM), neonatal sex, mode of birth, APGAR scores at 5 and 10 min, and umbilical arterial pH. Furthermore, anthropometric measurements at birth and at discharge were made, including weight, length, and head circumference.

### 2.5. Nutritional Management

Enteral and parenteral nutritional management was evaluated in all preterm infants during the hospital stay, and the nutritional support was provided according to ESPGHAN guidelines [41,42]. The mother’s own milk (MOM) or single-donor human milk, if MOM was unavailable, was initiated directly after birth. Feedings were advanced by 20–30 mL per kilogram per day. Human milk fortification was initiated when the infant reached 100 mL per kilogram per day using FM 85 (Nestle, Vevey, Switzerland) in infants with a gestational age greater than 26 weeks, or Humavant +6 (Prolacta Bioscience, California, United States of America) in infants with a gestational age below 26 weeks. Parenteral nutrition was initiated directly after birth and was discontinued once enteral intake reached 140–160 mL per kilogram per day. The target macronutrient supply for parenteral nutrition was 3.5 g/kg per day for protein, 4.0 g/kg per day for lipids, and 8–12 mg/kg per minute for carbohydrates. The total volume of MOM received during the hospitalization was recorded.

### 2.6. Statistical Analysis

Continuous data are described as the median and interquartile range (IQR), accordingly, and categorical variables are specified as the number and percent of patients for each category. Spearman’s rank correlation test was used to assess the correlation between sFlt-1/PlGF ratios, FFM z-score, FM z-score, and anthropometric parameters including weight, length, and head circumference at body composition measurement among women with HDP. Multivariable regression analysis was applied to examine the relationship between FFM, FM, and sFlt-1/PlGF ratio at term age in the two study groups (term and preterm infants). The model was adjusted for confounding factors including infant sex [43], age at birth [44], age at body composition measurement [44], SGA [45], antenatal steroid exposure [46], and illness severity score (SNAPPE-II) [47].

Missing data were handled using a complete-case approach. Study participants with missing values for a specific variable were excluded only from analyses that involved that variable. Given the small proportion of missing data (<5%), no imputation was performed, and as this was an exploratory analysis, no power calculation was conducted.

For statistical analysis, R version 4.2.1. (R Foundation for Statistical Computing, Vienna, Austria) and GraphPad Prism 8 (Graphpad Software, La Jolla, CA, USA) were utilized. Statistical significance was specified as a two-sided *p*-value of *p* < 0.05, and statistical analyses were conducted in collaboration with statistician Nicholas Longford.

## 3. Results

**Figure 1:**
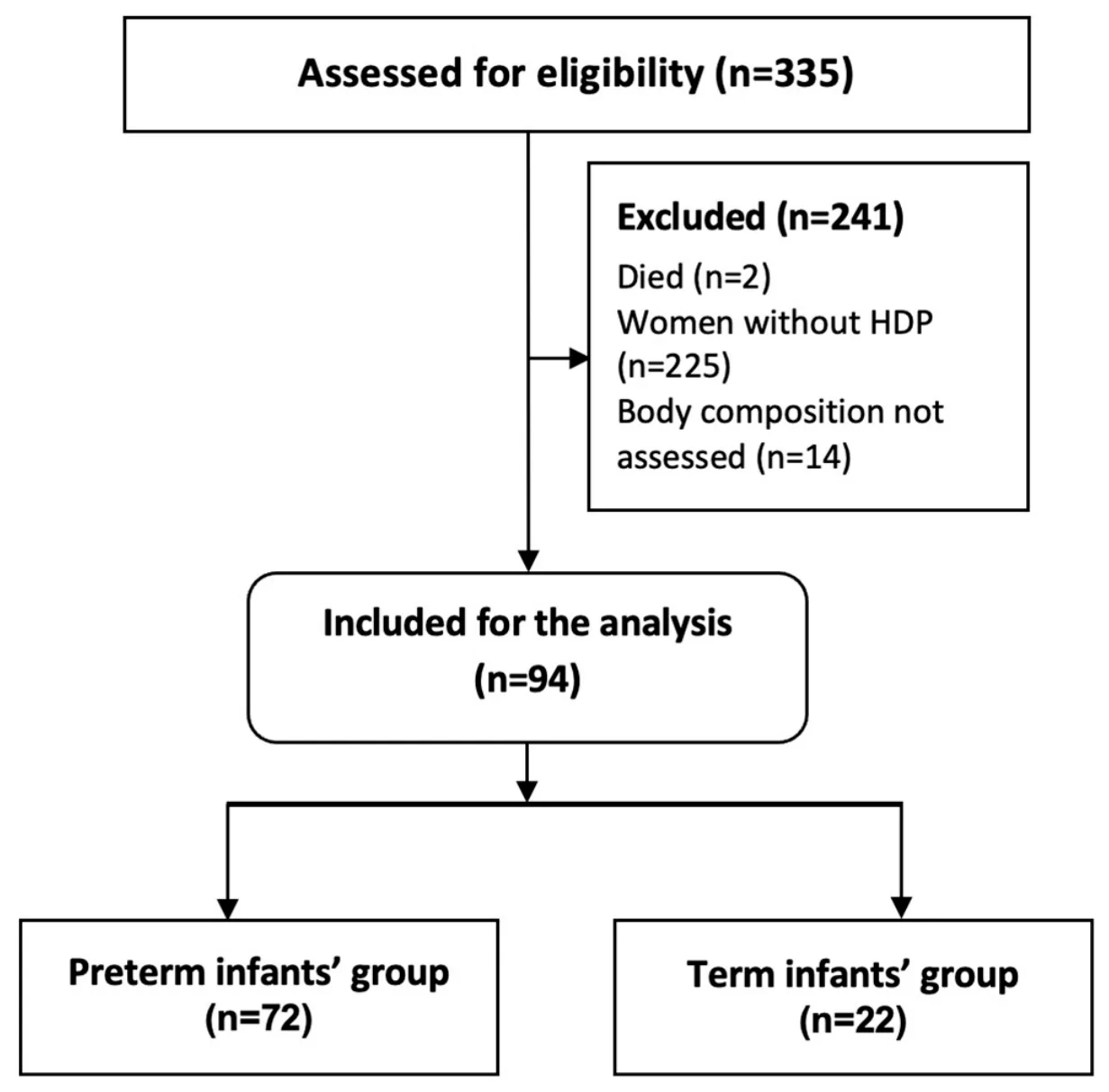
Overview of participants assessed for eligibility and term/preterm birth, including exclusion criteria.

A total of 335 infants were screened during the study period, 94 of which were included. Two preterm infants were excluded because they died during the hospital stay (one due to pulmonary hypertension crisis and one due to sepsis), and an additional 225 were excluded because they were born to mothers without HDP. In 14 other infants, body composition was not assessed, because of hemodynamic instability (*n* = 2), device error (*n* = 2), parental refusal (*n* = 3), or loss to follow-up (*n* = 7). An overview is shown in Figure 1.

### 3.1. Preterm Infants’ Group

Baseline characteristics and short-term outcome parameters of preterm infants are presented in Table 1. The median gestational age was 27.6 (IQR: 26.6, 30.5) weeks, the median birth weight was 850 (IQR: 660, 1585) grams, and the median birth length was 35.5 (IQR: 31.5, 43.0) cm. The proportion of infants born SGA was substantially higher and anthropometric parameters including z-scores for weight, length, and head circumference were substantially lower in the group with preterm infants compared to that with term infants (Table 1).

Levels of sFlt-1 and PlGF and the sFlt-1/PlGF ratio are shown in Table 1. As expected, maternal angiogenic markers were substantially elevated in mothers who delivered preterm infants than those who delivered term infants. The proportion of mothers with an sFlt-1/PlGF ratio <38 was substantially lower in the preterm infant group than in the term infant group.

A summary of the body composition measurements and anthropometric parameters at term-equivalent age is displayed in Table 2. Specifically, among infants with preterm birth, FM was 669 (IQR: 465, 887) grams, FFM was 2411 (IQR: 2049, 2699) grams, the FM z-score was −2.28 (IQR: −2.97, −1.82), and the FFM z-score was −3.89 (IQR: −4.56, −3.27).

The enteral and parenteral nutritional parameters are presented in Table 3. The median parenteral duration was 14 days (IQR: 10, 18) and 55.4% of infants exclusively received their mother’s own milk at discharge. Macronutrient intakes of protein, lipids, and carbohydrates are presented in Table 3.

**Figure 2:**
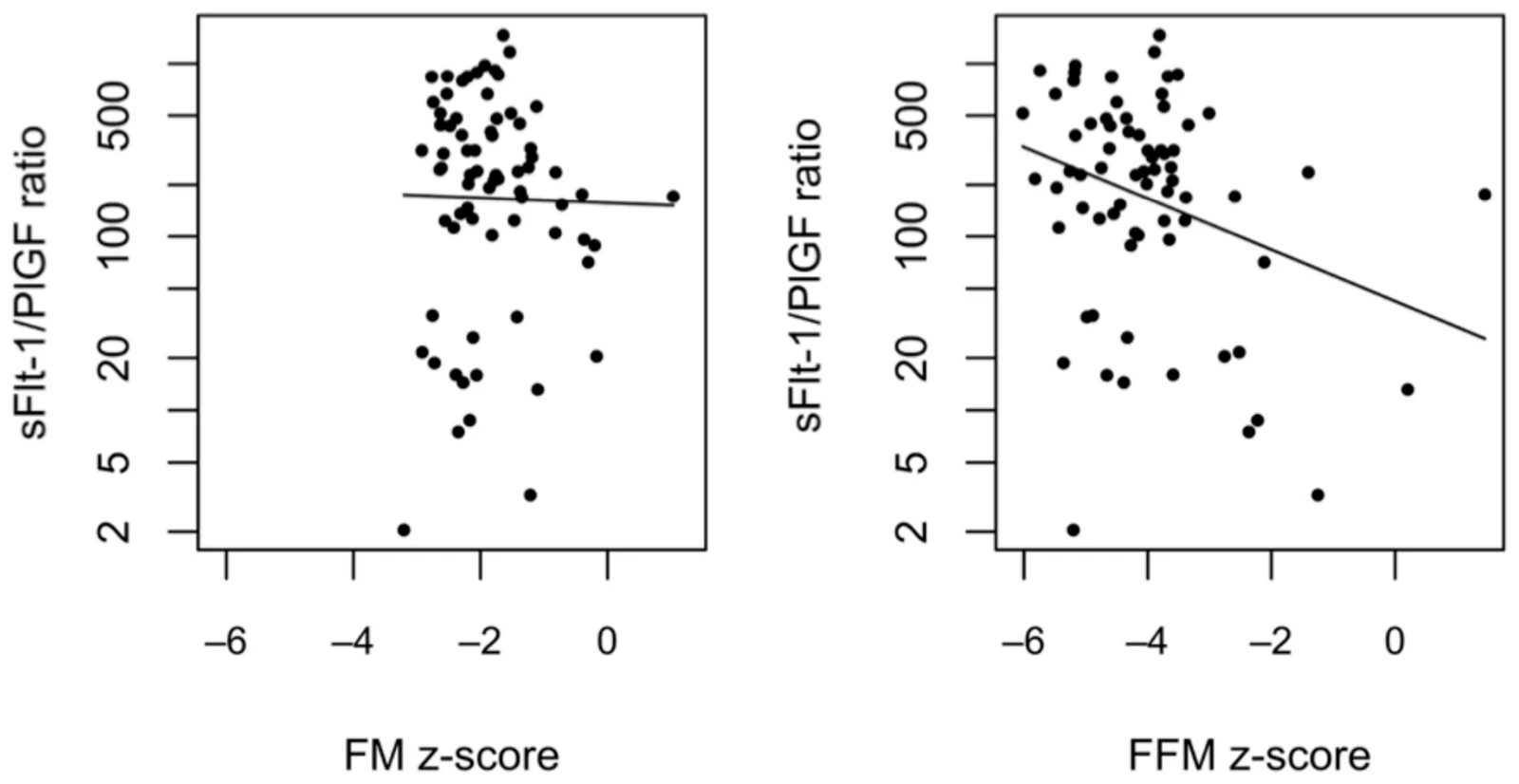
Correlation of sFlt-1/PlGF ratio with fat mass (FM) z-score and fat-free mass (FFM) z-score in preterm infants.

Higher sFlt-1/PlGF ratios were significantly correlated with lower FFM z-scores (rs = −0.408; *p* = 0.004) but not with FM z-scores (rs = −0.189; *p* = 0.21) at term-equivalent age in the preterm birth group (Figure 2).

sFlt-1/PlGF ratios were not significantly correlated with weight (rs = −0.199; *p* = 0.19), length (rs = −0.155; *p* = 0.31), and head circumference z-scores (rs = −0.158; *p* = 0.32) at the time of body composition measurement in the preterm infant group.

The linear regression model revealed that the sFlt-1/PlGF ratio was independently and significantly associated with a lower FFM z-score (median: −0.10, 95% CI: −0.2, 0.00; *p* = 0.036). Antenatal steroid exposure (median: 0.11, 95% CI: −0.75, 0.97; *p* = 0.79), sex (median: 0.43, 95% CI: −0.23, 1.10; *p* = 0.19), gestational age at birth (median: 0.09, 95% CI: −0.11, 0.28; *p* = 0.38), SGA (median: 0.29, 95% CI: −0.42, 0.98; *p* = 0.42), gestational age at body composition measurement (median: 0.07, 95% CI: −0.72, 0.21; *p* = 0.43), SNAPPE-II score (median: 0.01, 95% CI: −0.01, 0.04; *p* = 0.27), and the dichotomized maternal sFlt-1/PlGF ratio <38 vs. ≥38 (median: 0.50, 95% CI: −0.40, 1.40; *p* = 0.26) were not significantly associated with FFM z-score. FM z-scores were not significantly associated with any confounding factors (*p* < 0.05).

### 3.2. Term Infants’ Group

The median gestational age at birth was 39.1 weeks (IQR: 38.3, 40.0). None of the assessed short-term outcome parameters were observed in the term infant study group (Table 1).

At term-equivalent age, the median FM was 446 (IQR: 340, 613) g, the median FFM was 2722 (IQR: 2426, 3200) g (Table 2), the FM z-score was 1.38 (IQR: −0.85, 0.79), and the FFM z-score was −1.07 (IQR: −2.30, 0.28). In term infants, sFlt-1/PlGF ratios were not significantly correlated with FFM (rs = −0.040; *p* = 0.86), FM z-scores (rs = −0.186; *p* = 0.41), weight (rs = −0.191; *p* = 0.10), length (rs = 0.493; *p* = 0.12), and head circumference z-scores (rs = 0.495; *p* = 0.2) at the time of body composition measurement.

## 4. Discussion

This secondary analysis of a prospective cohort study investigated the relationship between maternal angiogenic markers (sFlt-1 and PlGF) in women with HDP and neonatal growth and body composition in their offspring. Body composition and anthropometric growth parameters were assessed at term-equivalent age in preterm infants and in term infants. Higher sFlt-1/PlGF ratios were significantly associated with lower FFM z-scores in preterm infants, but not in term infants. sFlt-1/PlGF ratios were not significantly associated with FM z-scores and anthropometric parameters, including weight, length, and head circumference at term-equivalent age, in either preterm or term infants.

These findings align with previous evidence that the sFlt-1/PLGF ratio is not only a well-established marker of HDP severity during pregnancy but is also associated with adverse effects on offspring body composition and growth, particularly in preterm infants [48].

The sFlt-1/PlGF ratio in women with HDP represents a key contributor to maternal morbidity and serves as an important clinical indicator of the disease [12,49,50]. Increasing evidence suggests that an imbalance in angiogenic markers, particularly the sFlt-1/PlGF ratio, constitutes a robust and reliable biomarker for both the diagnosis and stratification of disease severity [12,51,52]. The sFlt-1/PlGF ratio has been demonstrated to reflect the underlying pathophysiological process of placental dysfunction and endothelial disturbance [52,53]. In addition to maternal complications, HDP is well-established to adversely affect fetal growth and development, typically resulting in fetal growth restriction (FGR) and preterm birth. Despite extensive research on diagnostic applications, the potential impact of the sFlt-1/PlGF ratio on postnatal growth trajectories and body composition remains insufficiently explored. This lack of evidence is particularly relevant in preterm infants, as growth faltering is linked to impaired neurodevelopment and constitutes a critical determinant of neonatal morbidity. Therefore, the aim of this study was to investigate the association between angiogenic markers and neonatal growth patterns, including the assessment of body composition. In this context, particular attention was given to FFM, as reduced FFM at term-equivalent age has previously been associated with adverse neurodevelopmental outcomes extending into early childhood, up to 3 years of age in preterm born infants [22].

Angiogenic imbalance in placental insufficiency directly impacts fetal nutrient and oxygen delivery [26,54]. Increased sFlt-1/PlGF ratios are linked to SGA in infants, which is associated with prolonged parenteral nutrition, feeding intolerance, prolonged hospital stay, and impaired neurodevelopment [55]. Angiogenic imbalance leads to a disruption in placental vascular development by dysregulating VEGF pathways, causing a reduction in placental function that manifests as reduced fetal skeletal muscle growth and decreased FFM [27]. However, the effect of maternal angiogenic markers on neonatal morbidities and body composition including muscle growth has not been well-investigated so far.

The current study suggests that the sFlt-1/PlGF ratio might have long-term effects on fetal growth as well as on neonatal growth and body composition. This hypothesis is in line with the concept that placental dysfunction may contribute to early metabolic programming, affecting not only fetal growth but also postnatal body composition. In our study, a correlation between FFM z-scores and angiogenic markers was only seen in preterm infants and not in infants born at term. As the sFlt-1/PLGF ratio reflects HDP severity, a possible explanation for this might be that mothers of infants born at term were less severely affected than of newborns born preterm. This is supported by the observation that the sFlt-1/PlGF ratio was significantly higher in preterm infants (median: 232; IQR: 103, 464) in comparison to term infants (median: 33; IQR: 14, 48); (*p* < 0.001). Furthermore, the proportion of mothers with an sFlt-1/PlGF ratio <38 was substantially lower in the preterm infant group (19.4%) in comparison to the term infant group (63.6%).

Lower FFM z-scores have been consistently associated with adverse neurological outcomes in preterm infants [22,56,57]. Recent studies have shown that FFM at term-equivalent age is independently correlated with neurodevelopmental outcome up to three years of age [22]. Particularly, substantial early gains in FFM but not in FM are correlated with better cognitive outcomes [56]. Similarly, FFM z-scores at discharge are independently associated with neurologic outcome at 2 years of age, irrespective of gestational age, sex, and birth weight z-score [58]. These associations are likely mediated by brain growth, as higher FFM at term correlates with larger brain size measurements, including biparietal and bifrontal diameter and transverse cerebellar distance [59]. Previous studies have demonstrated that the nutritional management significantly influences growth and body composition, particularly in preterm infants [60,61,62,63]. Multiple factors contribute to these outcomes, including early aggressive nutritional strategies, higher protein intake, and optimization of energy provision [60]. Adequate protein intake has consistently been associated with improved lean mass accretion and enhanced brain growth [60,63,64]. Notably, infants born SGA exhibit reduced protein synthesis [65] and may therefore have higher protein requirements to support adequate catch-up growth and tissue accretion. This highlights the importance of tailored protein intake to the specific needs of this subgroup of preterm infants.

In this context, the observed association between higher sFlt-1/PlGF ratios and lower FFM might be indicative of a potential link between placental dysfunction and later neurodevelopmental outcomes, although this has to be established by further studies.

Of note, sFlt-1/PlGF ratios showed no significant correlation with FM in either group. Early FM gain has been associated with metabolic diseases including obesity, hypercholesterolemia, and diabetes in later life [66,67]. Current nutritional management might have limited impact on FM accretion and subsequent metabolic risk in later life. Further studies evaluating long-term metabolic outcomes are therefore needed.

Further research is required to determine whether standard nutritional management is sufficient in preventing growth faltering in these infants, since current standard feeding approaches and recommendations may not be sufficient [41]. Individualized nutritional management and aggressive and early nutritional strategies, including higher macronutrient provision and targeted fortification, may improve growth outcomes and body composition [68,69]. Previous studies have demonstrated that targeted fortification achieved by routinely measuring the nutrient content of human milk has a positive effect on growth and body composition [70]. Additionally, body composition measurement using air-displacement plethysmography has been shown to be a valuable tool for assessing nutritional status. Nevertheless, this method has some limitations, as measurements can only be performed when the infant is clinically stable without respiratory support at around term-equivalent age. Additionally, previous studies have shown that anthropometric parameters, especially weight gain, do not correlate with long-term neurodevelopmental outcomes [22]. Interestingly, in our study, sFlt-1/PlGF ratio was not significantly associated with weight, length, and head circumference z-scores at term-equivalent age in preterm and term infants. This evidence might support the concept that conventional anthropometric parameters may not adequately reflect early alteration in body composition or underlying nutritional status in high-risk neonates. Anthropometry mainly reflects the size and quantity of growth, whereas body composition reflects the quantity of growth, particularly FFM and FM.

There is a need to develop additional methods to evaluate nutritional status and guide nutritional management more effectively. In this context, bedside body composition assessment may represent a promising alternative, enabling earlier identification of growth faltering and inadequate body composition, especially FFM, and allowing timely adjustments of nutritional strategies before term-equivalent age. Further studies are necessary to investigate the clinical impact and utility, especially in preterm infants. Timely identification of high-risk groups and implementation of individualized nutritional strategies is crucial. Long-term studies are required to determine whether early alteration in body composition translates into long-term metabolic and cardiovascular as well as neurodevelopmental outcomes.

Furthermore, an important aspect highlighted by our data is the close interplay between obstetrics and neonatal care. Maternal angiogenic markers are primarily used in obstetrics to assess disease severity and guide pregnancy management in HDP [12,49,51]. Our study suggests that these markers may also have implications beyond pregnancy. Early identification of fetuses exposed to severe placental dysfunction may allow neonatologists to anticipate postnatal growth faltering and implement individualized nutritional strategies from birth. A multidisciplinary approach may help bridge the gap between prenatal risk assessment and postnatal nutritional strategies, ultimately improving growth and body composition as well as long-term neurodevelopmental outcomes.

This study has some limitations. It is a single-center observational study, and therefore, causal relationships cannot be established. Furthermore, the sample size is small, particularly in term infants. However, this is a prospective study, which strengthens the temporal relationship between exposure and outcome assessment. In addition, the sample size in preterm infants is relatively large, and body composition assessment was performed in 96% (305/319) of all study participants. Body composition was not assessed in only 14 infants, due to hemodynamic instability (*n* = 2), device error (*n* = 2), parental refusal (*n* = 3), or loss to follow-up (*n* = 7). Potential confounding of the observed link between FFM z-scores and angiogenic markers in preterm infants by other factors, such as gestational age and being SGA, cannot be ruled out. However, we accounted for potential confounders by conducting a multivariable regression analysis, adjusted for infants’ sex, gestational age at birth and measurement, SGA, antenatal steroid exposure, and the neonatal illness severity score (SNAPPE-II). Importantly, higher sFlt-1/PlGF ratios remained independently and significantly associated with lower FFM z-scores, after adjustment for these potential confounders. None of the other covariates was significantly associated with FFM z-scores. However, this study represents an exploratory secondary analysis of a prospective trial, so causal interferences cannot be drawn. In addition, the sample size was relatively small, especially for the term infant group, limiting statistical power. Larger prospective studies are warranted to confirm these findings. Nevertheless, this study demonstrates a potential association between maternal sFlt-1/PlGF ratio and neonatal body composition, particularly FFM. Further studies are needed to determine whether this association is causal.

## 5. Conclusions

To the best of our knowledge, this is the first study demonstrating that a higher maternal sFlt-1/PlGF ratio was significantly associated with lower FFM z-scores, measured by body composition at term-equivalent age in preterm infants. By contrast, the sFlt-1/PlGF ratio was not significantly associated with FM z-scores and anthropometric parameters, including weight, length, and head circumference, at term-equivalent age. FFM is well-known to depend largely on protein intake and accretion, and lower FFM has been associated with impaired brain development. Further research is required to determine whether individualized nutritional strategies could optimize early growth and body composition, which are crucial determinants of long-term neurodevelopmental outcomes in preterm infants.

## Figures and Tables

**Table 1:** Baseline characteristics and short-term outcomes of preterm (*n* = 72) and term neonates (*n* = 22).

	Preterm Infants	Term Infants
Patients, total number	72	22
Female, *n* (%)	40 (55.6)	13 (59.1)
Gestational age, weeks	27.6 [26.6, 30.5]	39.1 [38.3, 40.0]
Birth weight, gram	850 [660, 1585]	3195 [2928, 3763]
Z-score	−1.0 [−1.5, −0.5]	0.1 [−0.7, 0.7]
Birth length, cm	35.5 [31.5, 43.0]	50.0 [49.8, 53.1]
Z-score	−0.8 [−1.2, 0.1]	0.4 [−0.2, 1.4]
Birth head circumference, cm	25.5 [23.0, 30.0]	34.5 [33.0, 35.3]
Z-score	−0.4 [−1.2, 0.4]	0.2 [−0.6, 0.8]
Small for gestational age (<10 perc.), *n* (%)	27 (38.0)	1 (4.8)
Antenatal steroids, complete course, *n* (%)	51 (70.8)	0 (0.0)
Cesarean section, *n* (%)	66 (91.7)	7 (31.8)
APGAR, 5 min, points	9 [9, 9]	10 [9, 10]
APGAR, 10 min, points	9 [9, 10]	10 [10, 10]
Umbilical artery, pH	7.31 [7.28, 7.33]	7.27 [7.18, 7.34]
SFLT-1	9555 [6124, 16,065]	5407 [2995, 7389]
PLGF	47 [25, 92]	186 [132, 277]
SFLT-1/PLGF ratio	232 [103, 464]	33 [14, 48]
SFLI-1/PLGF score <38, *n* (%)	14 (19.4)	14 (63.6)
SNAPPE-II score	13 (0, 38)	−
Necrotizing enterocolitis stage ≥II, *n* (%)	3 (4.2)	0 (0.0)
Intraventricular hemorrhage grade III–IV, *n* (%)	2 (2.8)	0 (0.0)
Retinopathy of prematurity grade II–IV, *n* (%)	4 (5.6)	0 (0.0)
Bronchopulmonary dysplasia, *n* (%)	7 (9.7)	0 (0.0)

Data are presented in median and IQR (interquartile range), as well as numbers and percentages. HDPs: hypertensive disorders of pregnancy; SFLT-1: soluble Fms-like tyrosine kinase; PLGF: placental growth factor; SNAPPE-II: Score for Neonatal Acute Physiology with Perinatal Extension-II.

**Table 2:** Body composition and anthropometric measurements of preterm and term infants at term-equivalent age.

	Preterm Infants	Term Infants
Patients, total number	72	22
Age at measurement, weeks	40.5 [38.0, 42.6]	41.1 [39.3, 42.1]
FM, gram	669 [465, 887]	446 [340, 613]
FFM, gram	2411 [2049, 2699]	2722 [2426, 3200]
FM, percentage	21.1 [18.3, 26.4]	13.2 [10.5, 18.7]
FFM, percentage	79.0 [73.6, 81.7]	86.8 [81.3, 89.6]
FM, z-score	−2.28 [−2.97, −1.82]	1.38 [−0.85, 0.79]
FFM, z-score	−3.89 [−4.56, −3.27]	−1.07 [−2.30, 0.28]
Weight, cm	3102 [2517, 3477]	3078 [2892, 3692]
z-score	−1.3 [−1.9, −0.6]	−0.6 [−1.4, 0.5]
Length, cm	49.0 [47.0, 54.0]	50.0 [49.8, 53.1]
z-score	−1.2 [−2.3, −0.1]	0.4 [0.1, 1.5]
Head circumference, cm	35.0 [33.5, 36.0]	34.5 [33.0, 36.0]
z-score	−0.7 [−1.5, 0.1]	0.0 [−0.9, 0.5]

Data are presented in median and IQR (interquartile range). HDPs: hypertensive disorders of pregnancy; FFM: fat-free mass; and FM: fat mass.

**Table 3:** Overview of parenteral and enteral nutritional parameters in preterm infants.

	Preterm Infants
Patients, total number	72
Parenteral nutrition, days	14 [10, 18]
Carbohydrates, mg/kg/min	9.0 [7.8, 9.3]
Protein, g/kg/day	2.8 [2.4, 3.2]
Lipids, g/kg/day	2.9 [2.5, 3.2]
Exclusively mother’s milk at discharge, (%)	55.4
Mother’s own milk, total volume in liters	10.47 [4.91, 16.78]

Data are presented in median and IQR (interquartile range) percentage (%).

## Data Availability

The data is available upon reasonable request to the corresponding author. The data are not publicly available due to privacy and ethical restrictions.

## Abbreviations

The following abbreviations are used in this manuscript:

BPD	bronchopulmonary dysplasia
FFM	fat-free mass
FM	fat mass
HDPs	hypertensive disorders of pregnancy
IQR	interquartile range
IVH	intraventricular hemorrhage
NEC	necrotizing enterocolitis
PlGF	placental growth factor
pPROM	preterm premature rupture of membranes
ROP	retinopathy of prematurity
sFlt-1	soluble fms-like tyrosine kinase-1
SGA	small for gestational age
